# Receptor Interaction Profiles of 4-Alkoxy-3,5-Dimethoxy-Phenethylamines (Mescaline Derivatives) and Related Amphetamines

**DOI:** 10.3389/fphar.2021.794254

**Published:** 2022-02-09

**Authors:** Karolina E. Kolaczynska, Dino Luethi, Daniel Trachsel, Marius C. Hoener, Matthias E. Liechti

**Affiliations:** ^1^ Division of Clinical Pharmacology and Toxicology, Department of Biomedicine, University Hospital Basel and University of Basel, Basel, Switzerland; ^2^ Center for Physiology and Pharmacology, Institute of Pharmacology, Medical University of Vienna, Vienna, Austria; ^3^ ReseaChem GmbH, Burgdorf, Switzerland; ^4^ Neuroscience Research, pRED, Roche Innovation Center Basel, F. Hoffmann-La Roche Ltd, Basel, Switzerland

**Keywords:** phenethylamine, psychedelic, mescaline, scalines, 3C-scalines, fluorination

## Abstract

3,4,5-Trimethoxyphenethylamine (mescaline) is a psychedelic alkaloid found in peyote cactus. Related 4-alkoxy-3,5-dimethoxy-substituted phenethylamines (scalines) and amphetamines (3C-scalines) are reported to induce similarly potent psychedelic effects and are therefore potential novel therapeutics for psychedelic-assisted therapy. Herein, several pharmacologically uninvestigated scalines and 3C-scalines were examined at key monoamine targets *in vitro*. Binding affinity at human serotonergic 5-HT_1A_, 5-HT_2A_, and 5-HT_2C_, adrenergic α_1A_ and α_2A_, and dopaminergic D_2_ receptors, rat and mouse trace amine-associated receptor 1 (TAAR1), and human monoamine transporters were assessed using target specific transfected cells. Furthermore, activation of human 5-HT_2A_ and 5-HT_2B_ receptors, and TAAR1 was examined. Generally, scalines and 3C-scalines bound with weak to moderately high affinity to the 5-HT_2A_ receptor (*K*_i_ = 150–12,000 nM). 3C-scalines showed a marginal preference for the 5-HT_2A_ vs the 5-HT_2C_ and 5-HT_1A_ receptors whereas no preference was observed for the scalines. Extending the 4-alkoxy substituent increased 5-HT_2A_ and 5-HT_2C_ receptors binding affinities, and enhanced activation potency and efficacy at the 5-HT_2A_ but not at the 5-HT_2B_ receptor. Introduction of fluorinated 4-alkoxy substituents generally increased 5-HT_2A_ and 5-HT_2C_ receptors binding affinities and increased the activation potency and efficacy at the 5-HT_2A_ and 5-HT_2B_ receptors. Overall, no potent affinity was observed at non-serotonergic targets. As observed for other psychedelics, scalines and 3C-scalines interacted with the 5-HT_2A_ and 5-HT_2C_ receptors and bound with higher affinities (up to 63-fold and 34-fold increase, respectively) when compared to mescaline.

## 1 Introduction

**FIGURE 1:**
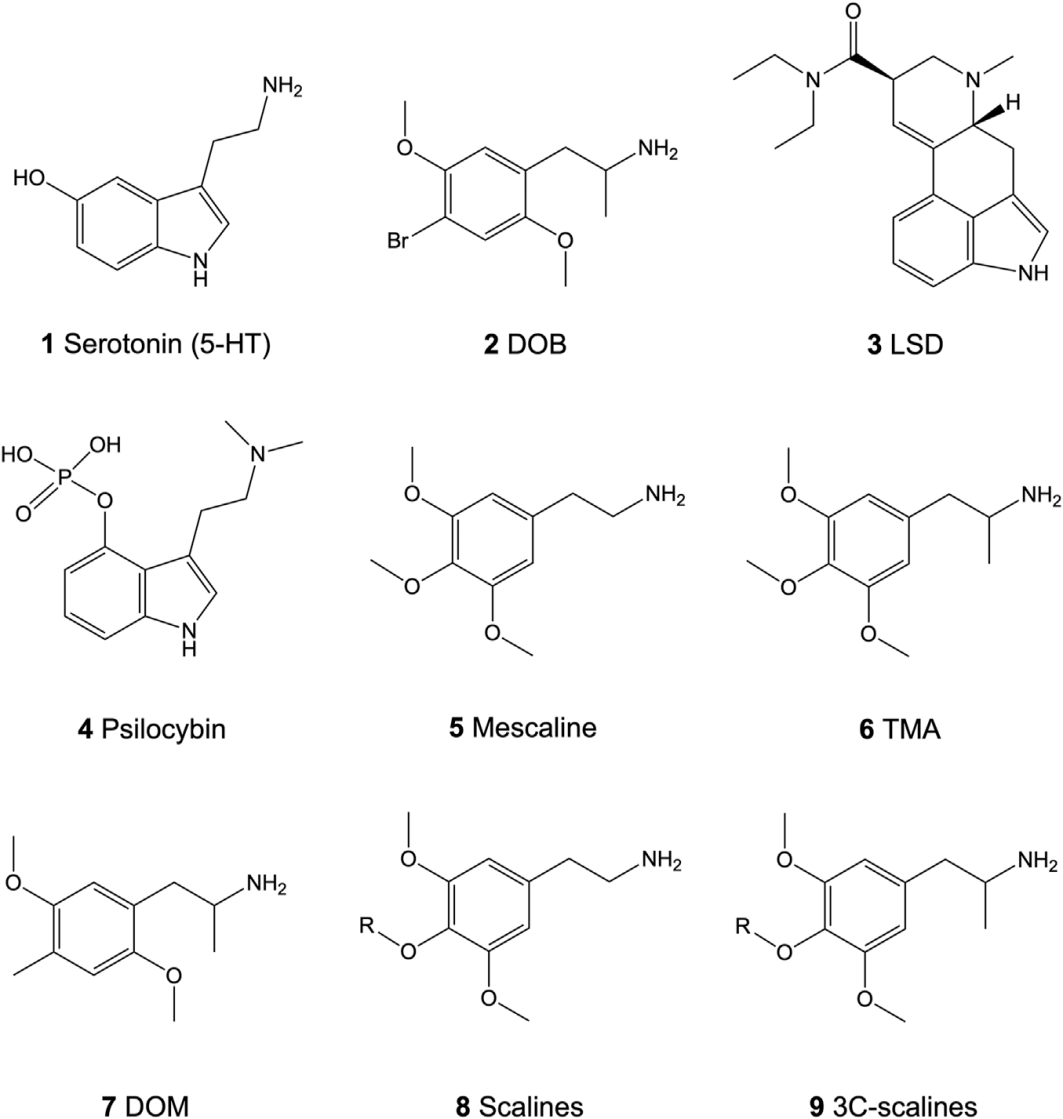
Chemical structures of the neurotransmitter serotonin (5-HT; **1**) and the psychedelics 4-bromo-2,5-dimethoxyamphetamine (DOB; **2**), lysergic acid diethylamide (LSD; **3**), psilocybin (**4**), 3,4,5-trimethoxyphenethylamine (mescaline; **5**), 3,4,5-trimethoxyamphetamine (TMA; **6**), and 2,5-dimethoxy-4-methyl-amphetamine (DOM; **7**), and the core structure of the 4-alkoxy-3,5-dimethoxyphenethylamines (scalines; **8**) and 4-alkoxy-3,5-dimethoxyamphetamine (3C-scalines; **9**).

Serotonin [5-hydroxytryptamine, 5-HT (**1**; Figure 1)] modulates vital central nervous system processes like appetite, sexual activity, memory, attention, or sleep through interactions with various 5-HT receptors (G protein-coupled receptors except for 5-HT_3_ receptors) (Berger et al., 2009; Pithadia and Jain 2009). Altered 5-HT modulation can lead to several psychiatric conditions like anxiety, depression, or schizophrenia (Rapport et al., 1948). Widely distributed in the central nervous system, the 5-HT_2_ receptor subtype (5-HT_2A_, 5-HT_2B_, and 5-HT_2C_ receptors) is a key pharmacological target for therapeutic drugs including antidepressants, anxiolytics, and antipsychotics (Roth 2011). Due to the lack of selectivity, however, identifying the various roles of each receptor subtype is difficult. In recent years, this issue has been tackled by the synthesis of selective ligands for each receptor isoform. Ligands which show high affinity-binding at the 5-HT_2_ receptor family but are devoid of subtype selectivity include substituted phenethylamines like 4-bromo-2,5-dimethoxyamphetamine (DOB; **2**; Figure 1) (Glennon et al., 1992; Monte et al., 1996; Chambers et al., 2001; Chambers et al., 2002). The 5-HT_2A_ and, albeit to a lesser extent, the 5-HT_2C_ receptor isoforms are both involved in the induction of psychedelic effects associated with classical psychedelics (Figure 1) like lysergic acid diethylamide (LSD; **3**) or psilocybin (**4**) as well as novel derivatives thereof (Glennon et al., 1992; Vollenweider et al., 1998; Chambers et al., 2002; Preller et al., 2016; Rickli et al., 2016; Preller et al., 2017; Luethi and Liechti 2020; Rudin et al., 2021). Both receptors mediate their effects *via* G_q_-protein-mediated activation of phospholipase C (PLC), which catalyzes the hydrolysis of phosphatidylinositol 4,5-biphosphate (PIP_2_) to diacyl glycerol (DAG) and inositol triphosphate (IP_3_). This then leads to protein kinase C activation and calcium release to initiate further downstream effects (Roth 2011). Moreover, G protein-independent signaling pathways mediated by β-arrestins are activated and involved in the receptor effects (Allen et al., 2008). In addition to similar signaling mechanisms, selective binding between the two receptors is difficult as the receptor isoforms share a high degree of sequence homology in both the agonistic and antagonistic ligand binding sites (Boess and Martin 1994; Trachsel et al., 2009). For the past five decades, some natural and many synthetic phenethylamines have been examined for their 5-HT_2_ receptor binding affinities and psychoactive effects (Aldous et al., 1974; Barfknecht and Nichols 1975; Glennon et al., 1982; Trachsel 2003; Trachsel et al., 2013; Rickli et al., 2015; Luethi et al., 2018b; Eshleman et al., 2018; Kolaczynska et al., 2019; Luethi et al., 2019; Luethi and Liechti 2020; Rudin et al., 2021). The large family consisting of more than hundred psychedelics includes 3,4,5-trimethoxyphenethylamine (mescaline; **5**) as the prototypical natural lead structure. Psychedelic phenethylamines can be classified into three distinct groups, based on their aryl substitution pattern: the 2,4,5-trisubstituted, the 2,4,6-trisubstituted, and the 3,4,5-trisubstituted compounds. For all three classes, some of the most active compounds contain two methoxy (MeO) groups allocated at the 2- or 3-position and at the 5- or 6-position. Modifications at the crucial 4-position may include small lipophilic substituents such as a Cl, Br, I, MeO, or methyl group, or larger lipophilic substituents such as a propylthio or a methallyloxy group. Currently, the *in vitro* and *in vivo* data available are mostly obtained from 2,4,5-trisubstituted derivatives [extensively reviewed in (Trachsel et al., 2013)].

Mescaline (**5**) was discovered as a natural ingredient of the psychoactive cactus *peyote* and identified as the principle pharmacological agent as early as in 1897 by Arthur Heffter (1898). Psychedelic doses of mescaline lie in the range of 180–360 mg or higher (Shulgin and Shulgin 1991). Mescaline’s α-methyl congener 3,4,5-trimethoxyamphetamine (TMA; **6**) was first synthesized in 1947 by P. Hey (1947) with active doses lying in the range of 100–200 mg (Shulgin and Shulgin 1991). Thus far, the thorough investigation of the structure-activity relationship (SAR) of 3,4,5-trisubstituted phenethylamines has been slow largely due to early reports of their relatively weak human potencies. The focus shifted even more to 2,4,5-trisubstituted derivatives when Alexander Shulgin discovered that some of these substances are active at doses well below 10 mg [e.g., DOB (**2**): 1–3 mg or 2,5-dimethoxy-4-methylamphetamine (**7**); DOM: 3–10 mg] (Shulgin and Shulgin 1991). None of the more than three dozen of 3,4,5-trisubstituted phenethylamines and related amphetamines predominantly investigated by Shulgin have proven to be fully active at doses below 20 mg (Shulgin and Shulgin 1991). Moreover, the few performed *in vitro* studies indicated a markedly lower affinity at the 5-HT_2A/2C_ receptors for 3,4,5-trisubstituted derivatives (scalines and 3C-scalines) compared to 2,4,5-trisubstituted phenethylamines. Retrospectively, however, this somewhat unwarranted focus towards the 2,4,5-series may be based on somewhat overhasty and generalized assumptions. Comparing human potencies of 2,5-dimethoxy- and 3,5-dimethoxyphenethylamines including their α-methyl congeners bearing identical 4-substituents revealed a far less distinctive predominance in favor of the 2,4,5-class (Trachsel et al., 2013). Some of the 4-substituents even lead to more potent 3,4,5-trisubstituted derivatives compared to 2,4,5-trisubstituted derivatives. Moreover, there are still numerous 4-substituents remaining to be tested within the 3,4,5-series, which will allow further comparisons and conclusions.

2,4,6-trisubstituted derivatives are even less investigated. However, the available data suggests that there seems to be more shared SAR for these compounds with the 2,4,5 derivatives than with the 3,4,5-trisubstituted series. To be specific, while some of the 2,4,6-trisubstituted derivatives with identical 4-substituent are significantly less potent in human than the 2,4,5-series, the identical 4-substituents lead to the most potent derivatives in both series so far (e.g., 4-Br or 4-Me) (Shulgin and Shulgin 1991; Trachsel et al., 2013). The same could be observed for 5-HT_2A/2C_ receptor interactions (Chambers et al., 2002). Also, conformational restriction of the MeO groups in both the 2,4,5- and 2,4,6-series lead to increased *in vitro* and *in vivo* potencies (Monte et al., 1996; Chambers et al., 2002). This is in contrast to the 3,4,5-trisubstituted compounds, where conformational restriction of the MeO groups of the mescaline molecule towards dihydrobenzofurane and tetrahydrobenzodifurane moieties only slightly increased 5-HT_2A_/_2C_ receptor affinities. However, in contrast to mescaline, they failed to fully substitute in a drug discrimination experiment [training drug: LSD; **3** (Monte et al., 1997)]. The authors of that study concluded that the MeO groups of mescaline might need to be non-constrained in order to conformationally adapt when activating the 5-HT_2A_ receptor. Therefore, the 3,4,5-trisubstituted phenethylamines may show a somewhat different binding mode than 2,4,5- or 2,4,6-trisubstituted compounds, and their functional potency may be of more importance than mere affinity. Another significant structural modifier is the presence of an α-methyl (α-Me) group. This only has a small effect on binding affinity of 2,4,5-trisubstituted derivatives at 5-HT_2A_/_2C_ receptors for *racemic* α-Me containing derivatives (amphetamines), since they show similar affinity at the receptor when compared to their equivalent phenethylamine counterparts (Johnson et al., 1990; Glennon et al., 1992; Dowd et al., 2000; Parrish et al., 2005; Kolaczynska et al., 2019). *In vivo,* introduction of an α-Me group into the 2,4,5-series has noteworthy effects on, e.g., drug discrimination experiments (Glennon et al., 1988; Glennon 1991) or on head-twitch response (Halberstadt et al., 2020), where significantly higher potencies have been observed for *racemic* α-Me-containing substances. In humans, these α-Me derivatives display up to one order of magnitude increased potency and usually significantly prolonged duration of action compared to their phenethylamine counterparts (Shulgin and Shulgin 1991). This can, to some extent, be explained by an increase in hydrophobic properties and metabolic stability observed for the amphetamines due to monoamine oxidase inhibition by the α-Me group (Glennon et al., 1983; Nichols 1991; Glennon et al., 1992). A stronger intrinsic activity (i.e., maximal response produced when the receptor is bound and activated by the compound) observed for the amphetamines when compared to their phenethylamine counterparts may also explain these SAR since the intrinsic activity plays a key role at the receptor (Nichols et al., 1994; Parrish et al., 2005). It is important to note that the role of configuration of the chiral center in the 2,4,5-series has been extensively investigated in binding studies (Johnson et al., 1987; Sadzot et al., 1989; Parrish et al., 2005; Braden 2007), drug discrimination studies (Glennon 1991), and human experiments (Shulgin and Shulgin 1991). As an overall conclusion, the amphetamines with an R-configuration behaved as the more potent enantiomers (eutomers). They not only showed a higher affinity to the 5-HT_2A_ receptor but also a higher functional potency and functional efficacy (intrinsic activity) than the S-enantiomers. The few human data available revealed a 2-fold increased potency for the R-enantiomers in comparison to the corresponding racemates, and the S-enantiomers contributed only very little to the psychedelic properties. Hitherto, the effect of chirality caused by α-Me introduction on the psychopharmacology of scalines has not been studied.

**FIGURE 2:**
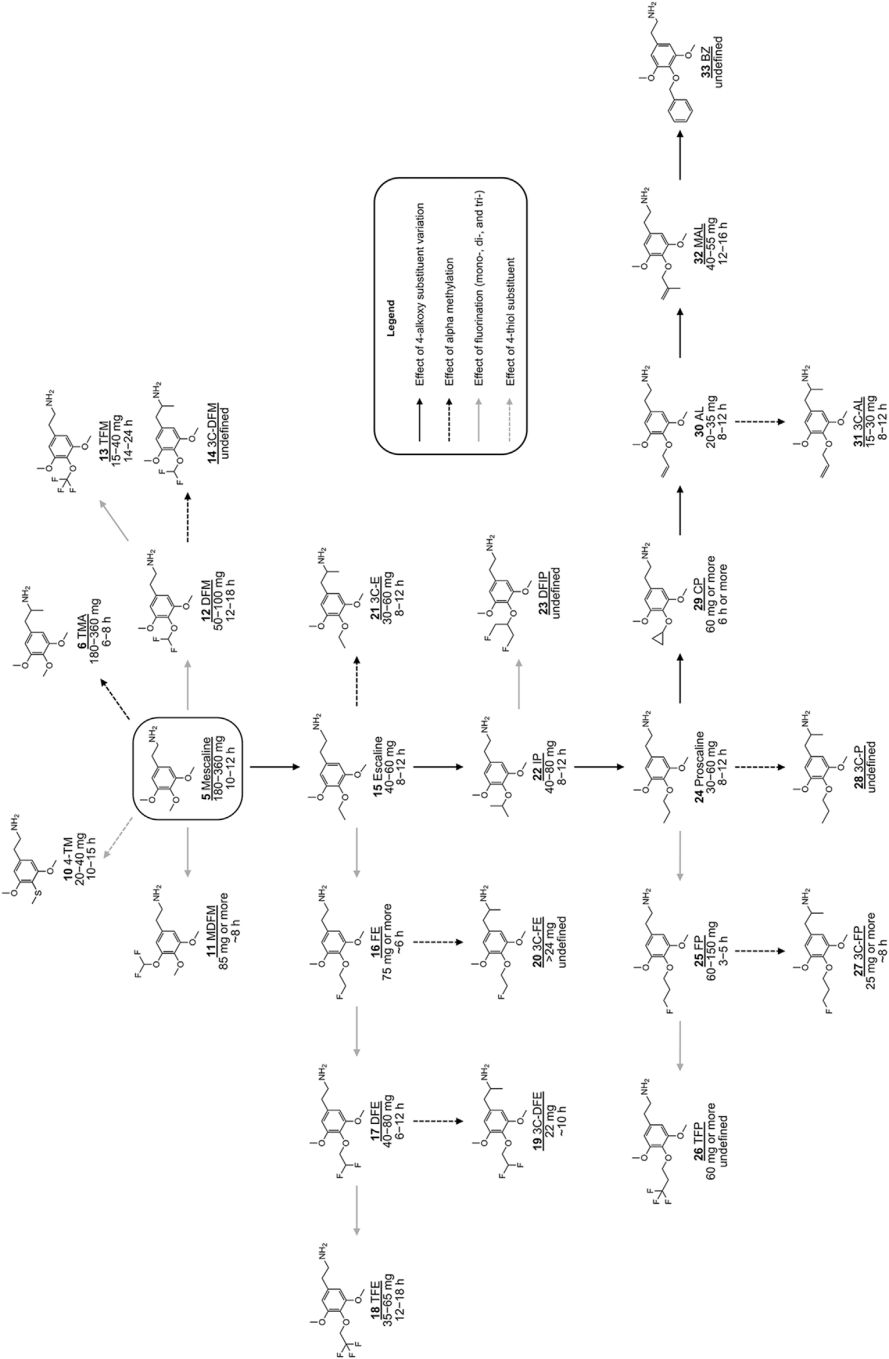
Chemical structures of various previously investigated scalines and 3C-scalines (Shulgin and Shulgin 1991; Shulgin and Shulgin 1997; Trachsel et al., 2013). Human oral doses and duration of action were taken from (Shulgin and Shulgin 1991; Shulgin and Shulgin 1997; Trachsel et al., 2013). Compounds tested *in vitro* in the present investigation are underlined.

Both animal and human observations with 2,4,5-trisubstituted derivatives are in strong contrast to what has been observed for scalines and 3C-scalines. The data available for comparison of 3,4,5-trisubstituted phenethylamine derivatives (Figure 1; **8**) with their α-Me congeners (Figure 1; **9**) showed an only marginal increase in dose potency and a comparable duration of action, with mescaline (**5**; 180–360 mg; 8–12 h) vs TMA (**6**; 100–250 mg; 6–8 h) being an exception (Shulgin and Shulgin 1991; Shulgin and Shulgin 1997; Trachsel et al., 2013). Moreover, 3,4,5-tri-*O*-substituted phenethylamines undergo a different amino oxidase-based metabolism than 2,4,5-tri-*O*-substituted phenethylamines (e.g., monoamine oxidase) (Clark et al., 1965). This might at least somewhat explain why the introduction of an α-Me group has only little influence on human doses of scalines vs 3C-scalines. With the data available so far, it remains difficult to draw solid conclusions or to apply existing SAR of one of the three different classes to another class. In spite of the many SAR available so far, the effects of 4-position substituents on 5-HT_2A/2C_ receptor interaction properties are not entirely understood and require further investigation. However, the overall experienced psychedelic effects are influenced several factors including agonist-to-antagonist transition, receptor activation potency, or interactions with additional targets (Ray 2010). As mentioned before, significant changes have been achieved with mescaline derivatives (Figure 2) bearing larger carbon chain lengths at the 4-alkoxy position. These derivatives include escaline (**15**), isoproscaline (IP; **22**), proscaline (**24**), allylescaline (AL; **30**), and methallylescaline (MAL; **32**). All of these compounds are significantly more potent than mescaline (**5**) in humans (effective doses ranging from 30 to 80 mg) and have a similar duration of action (8–12 h) (Shulgin and Shulgin 1991; Trachsel et al., 2013). Their psychedelic properties (i.e., the many different aspects of altered perception, mood and cognition, ego dissolution, and transcendence) seem to be changed significantly by modifying the chemical structure, at least based on interpreting the anecdotal data available so far (Trachsel et al., 2013).

The nomenclature for naming derivatives with a structural modification of the 4-substituent of mescaline (**5**) (i.e., the 4-MeO group) involves a common name in respect to their 4-substituent (Shulgin and Shulgin 1991; Trachsel et al., 2013). For example, the phenethylamine escaline (**15**) bears a 4-ethoxy group which can be shorted to a single letter, i.e., E. This single letter shortening can also be used to name several derivatives related to escaline, i.e fluoroescaline (FE; **16**), which can also be called FE. Furthermore, P stands for proscaline (**24**; 4-PrO), AL for allylescaline (**30**; 4-Allyloxy) etc. The α-Me group containing counterparts (amphetamines) are defined as 3C compounds, and this term is simply used as a prefix such as 3C-E (**21**) or 3C-AL (**31**) and so on (Shulgin and Shulgin 1991; Shulgin and Shulgin 1997; Trachsel et al., 2013).

A simple substitution of the 4-MeO group on mescaline (**5;** Figure 2) to a 4-S group, leads to 4-thiomescaline (4-TM; **10**), an analogue that has been shown to increase human potency 10-fold compared to **5** (active dose of **10** in humans = 20–40 mg) (Shulgin and Shulgin 1991). Introduction of fluorinated alkyloxy groups onto the 4-position of mescaline (**5**) has also led to derivatives with increased human potency when compared to **5** (Figure 2). These derivatives include difluoromescaline (DFM; **12**) and trifluromescaline (TFM; **13**), which have a 4-fold and > 9-fold increase in human potency, respectively. Both substances induce strong psychedelic effects and have significantly longer lasting effects than mescaline, with **13** being among the most potent mescaline-based derivatives synthesized to date (Trachsel 2012). Likewise, several fluorinated derivatives of the aforementioned 4-alkoxy analogues of mescaline have been synthesized (reviewed in (Trachsel 2012)), and initially pharmacologically investigated (Trachsel et al., 2013), e.g., compounds **5**–**33** (Figure 2).

In the light of the renewed interest in psychedelic substances in research and psychiatric therapy (Nichols 2016; Liechti 2017; Vollenweider and Preller 2020), investigating these derivatives is important to understand how certain structural modifications alter the way a derivative behaves at monoaminergic receptors and to gain further insight into the pharmacological properties of these derivatives. In the present investigation, we determined the receptor binding and activation properties of different mescaline derivatives and their α-Me containing counterparts at human serotonergic, adrenergic, and dopaminergic receptors, and at trace amine-associated receptor 1 (TAAR1). In addition, we explored the binding and inhibition potencies at human monoamine transporters.

## 2 Materials and Methods

### 2.1 Drugs

Full names and abbreviations of the test compounds are provided in Supplementary Table S1. The 3,5-dimethoxy-4-substituted phenethylamines (mescaline [**5**], MDFM [**11**], DFM [**12**], TFM [**13**], FE [**16**], DFE [**17**], TFE [**18**], IP [**22**], DFIP [**23**], FP [**25**], TFP [**26**], CP [**29**], MAL [**32**], BZ [**33**]) and the 3,5-dimethoxy-4-substituted amphetamines (TMA [**6**], 3C-DFM [**14**], 3C-DFE [**19**], 3C-FE [**20**], 3C-E [**21**], 3C-FP [**27**], 3C-P [**28**], 3C-AL [**31**]) were synthesized as racemates as previously described (Shulgin and Shulgin 1991; Trachsel 2002; Trachsel 2003; Trachsel et al., 2013), and provided as hydrochloride salts for pharmacological testing by ReseaChem (Burgdorf, Switzerland). Purity of all substances was >98%. [^3^H]serotonin (80.0 Ci/mmol) was purchased from Anawa (Zurich, Switzerland). [^3^H]dopamine (30.0 Ci/mmol) and [^3^H]norepinephrine (13.1 Ci/mmol) were obtained from Perkin-Elmer (Schwerzenbach, Switzerland).

### 2.2 Radioligand Receptor and Transporter Binding Assays

Radioligand binding affinity (*K*_i_) for monoamine receptors and transporters was assessed according to previously described methods (Luethi et al., 2018b). In short, different cell line derived membrane preparations overexpressing respective monoamine receptors (human genes with the exception of rat and mouse TAAR1) or transporter were briefly incubated with corresponding radiolabeled selective ligands at a concentration equal to the dissociation constant *K*_d_. The cell membrane preparations were obtained from Chinese hamster ovary cells (for hα_1A_ adrenergic receptor), Chinese hamster lung cells (for hα_2A_ adrenergic receptor) and HEK 293 cells (for h5-HT_1A_, h5-HT_2A_, h5-HT_2C,_ and hD_2_ receptors, TAAR1, and hNET, hDAT, and hSERT). The specific binding of radioligand to the target site was defined by measuring the difference between total binding and nonspecific binding (calculated in the presence of the respective receptor competitor in excess). This was used to measure the ligand displacement by the substances under investigation.

The following radioligands and their respective competitors were used: 0.90 nM [^3^H]8-hydroxy-2-(dipropylamine)tetralin (8-OH-DPAT) and 10 μM pindolol (h5-HT_1A_ receptor), 0.40 nM [^3^H]ketanserin and 10 μM spiperone (h5-HT_2A_ receptor), 1.4 nM [^3^H]mesulergine and 10 μM mianserin (h5-HT_2C_ receptor), 3.5 nM or 2.4 nM (rat or mouse isoform, respectively) [^3^H]RO5166017 and 10 μM RO5166017 (TAAR1), 0.11 nM [^3^H]prazosin and 10 μM chlorpromazine (hα_1A_ adrenergic receptor), 2 nM [^3^H]rauwolscine and 10 μM phentolamine (hα_2_ adrenergic receptor), 1.2 nM [^3^H]spiperone and 10 μM spiperone (dopaminergic hD_2_ receptor), 2.9 nM *N*-methyl-[^3^H]nisoxetine and 10 μM indatraline (hNET), 1.5 nM [^3^H]citalopram and 10 μM indatraline (hSERT), 3.3 nM [^3^H]WIN35,428 and 10 μM indatraline (hDAT).

### 2.3 Activity at the Serotonin 5-HT_2A_ Receptor

To assess the functional activity at the serotonin 5-HT_2A_ receptor, mouse embryonic fibroblasts (NIH-3T3 cells) expressing human 5-HT_2A_ receptor were seeded at a density of 70,000 cells per 100 μl in poly-D-lysine-coated 96-well plates according to methods previously described by Luethi, Trachsel et al. (2018). In brief, the NIH-3T3 cells were incubated in HEPES-Hank’s Balanced Salt Solution (HBSS) buffer (Gibco) for 1 h at 37°C. Subsequently, the plates were incubated with dye solution (100 μl/well) for 1 h at 37°C (fluorescence imaging plate reader [FLIPR] calcium 5 assay kit; Molecular Devices, Sunnyvale, CA, United States). Twenty-five microliter of test drugs diluted in HEPES-HBSS buffer composed of 250 mM probenecid were added to the plate online. Using nonlinear regression, the rise in fluorescence was measured and EC_50_ values were calculated from the concentration-response curves. The efficacy was calculated relative to 5-HT activity, which was defined as 100%.

### 2.4 Activity at the Serotonin 5-HT_2B_ Receptor

To assess the functional activity at the serotonin 5-HT_2B_ receptors, HEK 293 cells expressing the human 5-HT_2B_ receptor were seeded at a density of 50,000 cells per well in 96-well poly-D-lysine-coated plates overnight at 37°C, according to methods previously described by Luethi, Trachsel et al. (2018). In brief, the HEK 293 cells were incubated overnight at 37°C in high glucose Dulbecco’s modified Eagle’s medium (DMEM; Invitrogen, Zug, Switzerland), 10% fetal calf serum (non-dialyzed, heat-inactivated), 250 mg/L Geneticin and 10 ml/L PenStrep (Gibco). Using snap inversion, the growth medium was removed and 100 μl of calcium indicator Fluo-4 solution (Molecular Probes, Eugene, OR, United States) was added to each well for an incubation time of 45 min at 31°C. Thereafter, the Fluo-4 solution was removed (snap inversion) and subsequently an additional 100 μl of the Fluo-4 solution was added (incubation of 45 min at 31°C). Next, using the EMBLA cell washer, the cells were washed just before testing with HBSS and 20 mM HEPES and exposed to 100 μl of assay buffer. The plate was placed inside the FLIPR and 25 μl of test drugs diluted in assay buffer were added to the plate online. Using nonlinear regression, the rise in fluorescence was measured and EC_50_ values were calculated from the concentration-response curves. The efficacy was calculated relative to 5-HT activity, which was defined as 100%.

### 2.5 Activity at the Human TAAR1

To assess the functional activity at the human TAAR1, HEK 293 cells expressing recombinant human TAAR1 were grown in 250 ml falcon culture flasks containing 30 ml of high glucose DMEM [10% heat inactivated fetal calf serum, 500 μg/ml Geneticin (Gibco, Zug, Switzerland) and 500 μg/ml hygromycin B] at 37°C and 5% CO_2_/95% air, according to methods previously described by Luethi, Trachsel et al. (2018). Once 80–90% confluency was reached, the cells were collected by removing the medium, washing with PBS and then adding 5 ml of trypsin/EDTA solution for 5 min at 37°C. Next, 45 ml of medium was added and the entire mixture was transferred into a falcon tube. The tube was then centrifuged at room temperature for 3 min at 900 revolutions per minute (rpm). Next, the supernatant was removed in order to resuspend the remaining cell pellet in fresh medium to 5 × 10^5^ cells/ml. Hundred microliter of the cells was transferred into a 96-well plate (80,000 cells/well; BIOCOAT 6640, Becton Dickinson, Allschwil, Switzerland) and incubated for 20 h at 37°C.

For the cAMP assay, the aspirated medium was replaced with 50 μl PBS without Ca^2+^ and Mg^2+^ ions. Using snap inversion, the PBS was extracted and the plate was gently tapped against tissue. Next, 90 μl of Krebs-Ringer Bicarbonate buffer (KRB, Sigma-Aldrich) containing 1 mM IBMX was added and incubated for 60 min at 37°C and 5% CO_2_/95% air. Each test compound was examined in duplicate in a concentration range between 300 pM and 30 μM. A standard curve with a range of cAMP concentrations (0.13 nM–10 μM) was created for each 96-well plate. Each experiment was accompanied with a reference plate that included RO5256390, β-phenylethylamine and p-tyramine. The cells were exposed to either 30 μl of compound solution, 30 μl of β-phenylethylamine (as maximal response), or a basal control in PBS (containing 1 mM IBMX) for 40 min at 37°C. Next, under forceful shaking using black lids, the cells were exposed to 50 μl of 3× detection mix solution (composed of Ru-cAMP Alexa700 anti-cAMP antibody and lysis buffer) for 120 min at room temperature. Using the NanoScan reader (Innovate Optische Messtechnik, Berlin, Germany; 456 nm excitation wavelength; 630 and 700 nm emission wavelengths), the fluorescence was examined and the FRET signal was determined using the following equation; FRET (700 nm)- P × FRET (630 nm), where P = Ru (700 nm)/Ru (630 nm).

### 2.6 Monoamine Uptake Transporter Inhibition

To exclude activity of the scalines and 3C-scalines at monoamine transporters at pharmacologically relevant concentrations, a single high drug concentration was examined using HEK 293 cells stably transfected with the human serotonin, norepinephrine or dopamine transporters (hSERT, hNET, or hDAT, respectively) as previously described (Luethi et al., 2018a). In summary, the cells were cultured in DMEM (Gibco, Life Technologies, Zug, Switzerland) containing both 250 μg/ml Geneticin (Gibco) and 10% fetal bovine serum (Gibco). Once the cells were confluent (70–90%) they were detached and resuspended in KRB (Sigma-Aldrich, Buchs, Switzerland) at a density of 3 × 10^6^ cells/ml of buffer. For [^3^H]dopamine uptake experiments, the buffer additionally contained 0.2 mg/ml ascorbic acid. Hundred microliter of cell suspension per well was added to a round bottom 96-well plate. The cells were then incubated with 25 μl buffer containing the test drug (10 μM), vehicle control (0.1% dimethyl sulfoxide), or transporter-specific inhibitors [10 μM fluoxetine (SERT), 10 μM mazindol (DAT) or 10 μM nisoxetine (NET)] for 10 min by shaking on a rotary shaker (450 rpm) at room temperature. Uptake transport was initiated by adding [^3^H]serotonin, [^3^H]dopamine, or [^3^H]norepinephrine at a final concentration of 5 nM to the mixture. After 10 min, uptake transport was halted by the transfer of 100 μl of the cell mixture to 500 μl microcentrifuge tubes containing 50 μl of 3 M KOH and 200 μl silicon oil (1:1 mixture of silicon oil types AR 20 and 200; Sigma-Aldrich). The tubes were centrifuged for 3 min at 13,200 rpm, to allow the transport of the cells through the silicon oil layer into the KOH layer. The tubes were frozen in liquid nitrogen and the cell pellet was cut into 6 ml scintillation vials (Perkin-Elmer) containing 0.5 ml lysis buffer (1% NP-40, 50 mM NaCl, 0.05 M TRIS-HCl, 5 mM EDTA and deionized water). The samples were shaken for 1 h before 3 ml of scintillation fluid (Ultima Gold, Perkin Elmer, Schwerzenbach, Switzerland) was added. Monoamine uptake was then quantified by liquid scintillation counting on a Packard Tri-Carb Liquid Scintillation Counter 1900 TR. Nonspecific uptake in the presence of selective inhibitors was subtracted from the total counts.

### 2.7 Statistical Analysis

All calculations and analyses were performed using Prism 7.0a (GraphPad, San Diego, CA, United States). IC_50_ values of the radioligand binding were determined by calculating nonlinear regression curves for a one-site model using at least three independent 10-point concentration-response curves for each substance. The *K*_i_ values correspond to the dissociative constant for the inhibitor and were calculated using the Cheng-Prusoff equation. Nonlinear regression concentration-response curves were used to determine EC_50_ values for 5-HT_2A_ and 5-HT_2B_ receptor activation. Maximal activation activity (efficacy) is expressed relative to the activity of 5-HT, which was set to 100%. Monoamine uptake of four independent experiments was compared to control using 1-way ANOVA analysis of variance followed by a Dunett’s multiple-comparison test. Monoamine uptake of MDMA was included as comparison. Receptor affinity binding (*K*_i_) < 50 nM was defined as high affinity binding, *K*_i_ < 500 nM as moderate affinity binding, while *K*_i_ > 1,000 nM was defined as low affinity binding. Activation efficacy (max %) < 85% was defined as partial agonism while max % > 85% was defined as full agonism.

## 3 Results

### 3.1 Binding and Activation of the Serotonin Receptors

#### 3.1.1 5-HT_1A_ Receptors

The 5-HT receptor binding affinities and activation potencies of the examined derivatives are listed in Table 1. The classical psychedelics LSD and 2C-B were previously tested using the same assays and included for comparison (Rickli et al., 2016; Luethi et al., 2018b). Among the phenethylamines, mescaline, MDFM, DFM TFM, CP, MAL, and BZ (Figure 2, structures **5, 11, 12, 13, 29, 32** and **33**, respectively) were the only compounds that bound to the 5-HT_1A_ receptor, albeit only in the lower micromolar range (*K*_i_ = 1.6–6.7 μM). In contrast, none of the 3C-scalines (Figure 2, structures **6, 14, 19, 20, 21, 27, 28**, and **31**, respectively) bound to the 5-HT_1A_ receptor at the concentrations tested (*K*_i_ > 5,600 nM).

**TABLE 1:** Serotonin receptor binding affinities and activation potencies of 4-alkoxy-substituted 3,5-dimethoxyphenethylamines and amphetamines.

			h5-HT_1A_	h5-HT_2A_			h5-HT_2B_		h5-HT_2C_	Selectivity	
			Receptor binding	Receptor binding	Activation potency	Activation efficacy	Activation potency	Activation efficacy	Receptor binding	5-HT_2A_ vs. 5-HT_1A_	5-HT_2A_ vs. 5-HT_2C_
			*K*_i_ ± SD (nM)	*K*i ± SD (nM)	EC_50_ ± SD (nM)	max ± SD (%)	EC_50_ ± SD (nM)	max ± SD (%)	*K*_i_ ± SD (nM)		
			[^3^H]8-OH-DPAT	[^3^H]ketanserin					[^3^H]mesulergine		
**4-alkoxy-substituted 3,5-dimethoxyphenethylamines**											
** 5**	Mescaline*		6,700 ± 600	9,400 ± 2,100	^a^10,000 ± 1,800	^a^56 ± 15	>10,000		9,900 ± 2,800	0.7	1.1
** 11**	MDFM		1,600 ± 2,00	1,900 ± 6,00	190 ± 10	52 ± 4	200 ± 60	31 ± 19	1,500 ± 900	0.8	0.8
** 12**	DFM		2,500 ± 1,300	3,500 ± 910	1,500 ± 110	94 ± 3	>10,000		2,800 ± 230	0.7	0.8
**13**	TFM		2,200 ± 200	280 ± 100	280 ± 120	71 ± 31	88 ± 20	45 ± 6	290 ± 100	7.9	1.0
** 16**	FE**		>5,600	>12,000	5,700 ± 1,100	89 ± 8	2,300 ± 1,200	29 ± 7	5,700 ± 3,300	NA	>1
** 17**	DFE***		>5,600	2,900 ± 1,500	1,300 ± 600	44 ± 5	1,700 ± 1,000	25 ± 7	2,700 ± 200	<1	0.9
** 18**	TFE		>5,600	1,300 ± 500	960 ± 50	61 ± 2	210 ± 130	29 ± 14	1,200 ± 300	<1	0.9
** 22**	IP		>5,600	6,200 ± 2,600	1,900 ± 400	78 ± 11	>10,000		5,400 ± 1,900	>1	0.9
** 23**	DFIP		>5,600	>12,000			>10,000		>10,000	NA	NA
** 25**	FP****		>5,600	9,300 ± 2,300	4,500 ± 700	87 ± 10	>10,000		5,300 ± 1,400	<1	0.6
** 26**	TFP		>5,600	5,000 ± 1,700	4,900 ± 1,000	78 ± 6	>10,000		5,300 ± 900	>1	1.1
** 29**	CP		4,000 ± 100	4,300 ± 1,900	2,600 ± 500	86 ± 7	>10,000		5,600 ± 1,100	0.9	1.3
** 32**	MAL		5,100 ± 500	550 ± 190	79 ± 12	85 ± 5	>10,000		520 ± 150	9.3	0.9
** 33**	BZ		4,400 ± 200	150 ± 10	27 ± 8	77 ± 10	>10,000		440 ± 120	29	2.9
**4-alkoxy-substituted 3,5-dimethoxyamphetamines**											
**6**	TMA*		>5,600	>12,000	1700 ± 400	40 ± 6	>10,000		>10,000	NA	NA
** 14**	3C-DFM		>5,600	1,400 ± 900	76 ± 40	73 ± 6	150 ± 100	22 ± 7	3,700 ± 400	<1	2.6
** 19**	3C-DFE***		>5,600	1,500 ± 300	120 ± 20	95 ± 9	260 ± 30	22 ± 11	2,600 ± 1,400	<1	1.7
** 20**	3C-FE**		>5,600	12,000 ± 1700	120 ± 40	102 ± 16	800 ± 240	29 ± 6	8,400 ± 4,300	>1	>1
** 21**	3C-E		>5,600	3,700 ± 1500	160 ± 50	90 ± 4	95 ± 130	18 ± 13	4,900 ± 1,200	<1	1.3
** 27**	3C-FP****		>5,600	2,600 ± 1600	57 ± 2	62 ± 14	>10,000		4,400 ± 3,200	<1	1.7
** 28**	3C-P		>5,600	1,000 ± 460	160 ± 20	86 ± 0	>10,000		2,000 ± 1,000	<1	2.0
** 31**	3C-AL		>5,600	1,100 ± 350	190 ± 30	61 ± 9	>10,000		1,700 ± 800	<1	1.7
**Reference substances**											
	2C-B^b^		311 ± 46	6.9 ± 1.8	2.1 ± 0.8	92 ± 8	75 ± 14	52 ± 26	43 ± 4	45	6.2
**3**	LSD^b^		1.5 ± 0.4	5.3 ± 3.4	44 ± 14	73 ± 2	>10,000	NA	14 ± 3	0.28	2.6

*K*_i_ and EC_50_ values are given as nM (mean ± SD); activation efficacy (E_max_) is given as percentage of maximum ± SD.

Asterisks indicate corresponding pairs of derivatives with the same modifications. NA, not assessed.

a Data taken from Rickli et al. (2016).

b Data taken from Luethi et al. (2018b).

#### 3.1.2 5-HT_2A_ Receptors

The fluorinated and bulky substituted phenethylamines TFM (**13**), MAL (**32**), and BZ (**33**) bound relatively potently to the 5-HT_2A_ receptor in the submicromolar range (*K*_i_ = 150–550 nM). The remaining compounds (structures **11**, **12**, **17**, **18**, **22**, **25**, **26**, and **29**) bound in the micromolar range (*K*_i_ = 1,300–9,400 nM) with the exception of FE and DFIP (**16** and **23**; *K*_i_ > 12,000 nM). Compounds **5**, **11**, **13**, **17**, **18**, **22**, **26**, and **33** were 5-HT_2A_ receptor partial agonists with EC_50_ values in the range of 27–10,000 nM and activation efficacies of 44–78%. DFM (**12**), FE (**16**), FP (**25**), CP (**29**), and MAL (**32**) activated the 5-HT_2A_ receptor as a full agonists with EC_50_ values in the range of 79–5,700 nM and activation efficacy of 85–94%.

The amphetamines bound to the 5-HT_2A_ receptor in the micromolar range (structures **14, 19, 21, 27, 28,** and **31;** *K*_i_ = 1,000–3,700 nM) with the exception of TMA and 3C-FE (**6** and **20**; *K*_i_ > 12,000 nM). The amphetamines activated the receptor as with EC_50_ values in the range of 57–1,700 nM. Some amphetamines (**6**, **14**, **27**, and **31**) activated the receptor as partial agonists with activation efficacies of 40–73%. 3C-DFE (**12**), 3C-FE (**13**), 3C-E (**14**), and 3C-P (**31**) were full agonists at the 5-HT_2A_ receptor (activation efficacy = 86–102%).

#### 3.1.3 5-HT_2B_ Receptors

The fluorinated phenethylamines MDFM (**11**), TFM (**13**), and TFE (**18**) activated the 5-HT_2B_ receptor in the submicromolar range (EC_50_ = 88–210 nM), while FE (**16**) and DFE (**17**) activated the receptor in the micromolar range (EC_50_ = 1,700–2,300 nM). All of these compounds were relatively low efficacy partial agonists at the 5-HT_2B_ receptor (activation efficacy = 25–45%). The remaining phenethylamines did not activate the 5-HT_2B_ receptor (EC_50_ > 10,000 nM). The amphetamine derivatives 3C-DFM (**14**), 3C-DFE (**19**), 3C-FE (**20**), and 3C-E (**21**) activated the 5-HT_2B_ receptor in the submicromolar range (EC_50_ = 95–800 nM) as low efficacy partial agonists with activation efficacy in the range of 18–29%. The remaining amphetamine derivatives did not activate the 5-HT_2B_ receptor (EC_50_ > 10,000 nM).

#### 3.1.4 5-HT_2C_ Receptors

Most compounds bound to the 5-HT_2C_ receptor with micromolar affinity (*K*_i_ = 1,200–9,900 nM). Exception to this were the phenethylamines TFM (**13**), MAL (**32**), and BZ (**33**), which bound to the 5-HT_2C_ receptor with submicromolar affinity (*K*_i_ = 290–520 nM) and DFIP (**23**), which did not bind to the 5-HT_2C_ receptor (*K*_i_ > 10,000 nM).

### 3.2 Interactions With Non-Serotonergic Receptors and Monoamine Transporters

Monoamine receptor and transporter binding affinities are listed in Table 2. None of the examined compounds activated the human TAAR1 (EC_50_ > 10,000 nM). At the rat TAAR1, most phenethylamine derivatives bound within a micromolar range (*K*_i_ = 1,000–3,000 nM) with the exception of DFM (**12**), TFM (**13**), TFP (**26**), and BZ (**33**), which bound at submicromolar concentrations (*K*_i_ = 110–910 nM). FE (**16**), IP (**22**), and DFIP (**23**) did not bind to the rat TAAR1 at the concentrations tested (*K*_i_ > 4,000 nM). The amphetamine derivative 3C-DFM (**14**) bound with a *K*_i_ of 380 nM to the rat TAAR1. TMA (**6**), 3C-DFE (**19**), 3C-P (**28**), and 3C-AL (**31**) bound in the micromolar range (*K*_i_ = 3,200–3,900 nM); for 3C-FE (**20**), 3C-E (**21**), and 3C-FP (**27**) no binding was observed at the rat TAAR1 at examined concentrations (*K*_i_ > 4,700 nM).

**TABLE 2:** Non-serotonergic receptor and transporter binding affinities of 4-alkoxy-subsituted 3,5-dimethoxyphenethylamines and amphetamines.

			hTAAR1	rTAAR1	mTAAR1	hα_1A_	Hα_2A_	hD_2_	hNET	hDAT	hSERT
			EC50 ± SD(nM)	*K*_i_ ± (nM)	*K*_i_ ± (nM)	*K*_i_ ± (nM)	*K*_i_ ± (nM)	*K*_i_ ± (nM)	*K*_i_ ± (nM)	*K*_i_ ± (nM)	*K*_i_ ± (nM)
				[^3^H]RO5166017	[^3^H]RO5166017	[^3^H]prazosin	[^3^H]rauwolscine	[^3^H]spiperone	*N-methy*-[^3^H]nisoxetine	[^3^H]WIN35,428	[^3^H]citalopram
**4-alkoxy-subsituted 3,5 dimethoxyphenethylamines**										
**5**	Mescaline*		NA	3,000 ± 200	>4,200	>8,700	2,000 ± 300	>6,300	>9,700	>8,500	>7,500
**11**	MDFM		>10000	1,100 ± 0	3,400 ± 1,000	4,300 ± 400	1,300 ± 100	>6,300	>9,700	>8,500	>7,500
**12**	DFM		NA	880 ± 180	>4,200	8,000 ± 1,300	1,700 ± 310	>6,300	>9,700	>8,500	>7,500
**13**	TFM		>10000	170 ± 10	1,900 ± 300	3,200 ± 800	450 ± 60	>6,300	>9,700	>8,500	>7,500
**16**	FE**		NA	>4,000	>4,200	>8,700	3,700 ± 100	>6,300	>9,700	>8,500	>7,500
**17**	DFE**		NA	2,100 ± 200	>4,200	>8,700	2,700 ± 300	>6,300	>9,700	>8,500	>7,500
**18**	TFE		>10000	1,200 ± 0	>4,200	>8,700	2,300 ± 200	>6,300	>9,700	>8,500	>7,500
**22**	IP		NA	>4,700	>4,200	>8,700	1,200 ± 200	>6,300	>9,700	>8,500	>7,500
**23**	DFIP		>10000	>4,700	>4,200	>8,700	2,700 ± 400	>6,300	>9,700	>8,500	>7,500
**25**	FP****		>10000	1,700 ± 100	>4,200	>8,700	2,900 ± 500	>6,300	>9,700	>8,500	>7,500
**26**	TFP		>10000	910 ± 90	>4,200	>8,700	2,300 ± 300	>6,300	>9,700	>8,500	>7,500
**29**	CP		>10000	1,200 ± 100	>4,200	>8,700	1,200 ± 300	>6,300	>9,700	>8,500	>7,500
**32**	MAL		>10000	1,000 ± 200	3,900 ± 200	>8,700	1,500 ± 500	>6,300	>9,700	>8,500	>7,500
**33**	BZ		>10000	110 ± 10	2,400 ± 500	>8,700	2,300 ± 100	>6,300	>9,700	>8,500	>7,500
**4-alkoxy-subsituted 3,5 dimethoxyamphetamines**										
**6**	TMA*		>10000	3,200 ± 400	1,800 ± 100	>8,700	4,030 ± 580	>6,300	>9,700	>8,500	>7,500
**14**	3C-DFM*		>10000	380 ± 20	1,000 ± 200	>8,700	2,600 ± 0	>6,300	>9,700	>8,500	>7,500
**19**	3C-DFM***		NA	3,900 ± 800	>4,200	>8700	>5,100	>6,300	>9,700	>8,500	>7,500
**20**	3C-FM**		NA	>4,700	>4,200	>8,700	>5,100	>6,300	>9,700	>8,500	>7,500
**21**	3C-FM		NA	>4,700	>4,200	>8,700	>5,100	>6,300	>9,700	>8,500	>7,500
**27**	3C-FP****		NA	>4,700	2,800 ± 500	>8,700	>5,100	>6,300	>9,700	>8,500	>7,500
**28**	3C-P		>10000	3,400 ± 300	1,800 ± 0	>8,700	4,600 ± 500	>6,300	>9,700	>8,500	>7,500
**31**	3C-AL		NA	3,600 ± 400	3,300 ± 500	>8,700	>5,100	>6,300	>9,700	>8,500	>7,500
**Reference substance**										
	MDMA^a^		NA	370 ± 120	2,400 ± 1,100	>8,700	>5,100	>6,300	>9,700	>8,500	>7,500

*K*_i_ and EC_50_ values are given as nM (mean ± SD); activation efficacy (E_max_) is given as percentage of maximum ± SD.

Asterisks indicate corresponding pairs of derivatives with the same modifications. NA, not assessed.

a Data taken from Simmler et al. (2013).

At the mouse TAAR1, the phenethylamine derivatives MDFM (**11**), TFM (**13**), MAL (**32**), and BZ (**33**) bound in the micromolar range (*K*_i_ = 1,900–3,900 nM) while none of the remaining derivatives bound to the receptor (*K*_i_ > 4,200 nM). The amphetamine derivatives TMA (**6**), 3C-DFM (**14**), 3C-DFE (**19**), 3C-P (**28**), and 3C-AL (**31**) bound in the micromolar concentration range to the mouse TAAR1 (*K*_i_ = 1,000–3,300 nM); 3C-DFE (**19**), 3C-FE (**20**), and 3C-E (**21**) did not bind at examined concentrations (*K*_i_ > 4,200 nM).

At the adrenergic α_1A_ receptor, the phenethylamine derivatives MDFM (**11**), DFM (**12**), and TFM (**13**) were the only derivatives showing any affinities at tested concentrations (*K*_i_ = 3,200–8,000 nM). At the α_2A_ receptor, all phenethylamine derivatives bound in the micromolar range (*K*_i_ = 1,200–3,700 nM) except for TFM (**13**), which bound with moderate affinity (*K*_i_ = 450 nM). The only amphetamine derivatives that bound to the α_2A_ receptors were TMA (**6**), 3C-DFM (**14**), and 3C-P (**28**) (*K*_i_ = 2,600–4,600 nM).

None of the compounds examined bound to the dopaminergic D_2_ receptor (*K*_i_ > 6,300 nM) or any of the monoamine transporters (*K*_i_ > 7,500 nM). Furthermore, none of the investigated compounds significantly inhibited any of the monoamine uptake transporters (IC_50_ > 10,000 nM).

## 4 Discussion

### 4.1 5-HT Receptor Binding

Taken from the extensive SAR of 2,4,5-trisubstituted derivatives, small lipophilic substituents at the 4-position of 2,5-dimethoxy substituted phenethylamines and amphetamines lead to derivatives that have agonistic properties, while derivatives with large lipophilic substituents at the 4-position lead to antagonistic effects at the 5-HT_2A/2C_ receptors (Dowd et al., 2000). Furthermore, hydrophilic substituents at the 4-position attenuate 5-HT_2A_ receptor affinity and *in vivo* potency (Nelson et al., 1999; Braden 2007). In line with functional properties of ligands with lipophilic 4-substituents, a similar trend could be observed when reviewing the active doses of these compounds as psychedelics in man; when surpassing a certain steric bulkiness, compounds tend to lose their psychedelic properties (Shulgin and Shulgin 1991; Trachsel et al., 2013). Thus, smaller lipophilic 4-substituents not only yield agonists/partial agonists but also lead to the most potent psychedelics.

Thus far, the few *in vitro* investigated 3,4,5-trisubstituted phenethylamines and amphetamines have been shown to have the lowest 5-HT_2A_ receptor affinities among psychedelic phenethylamines (Monte et al., 1997; Parker et al., 2008; Trachsel et al., 2013) when compared to the 2,4,5-trisubsituted and 2,4,6-trisubsituted phenethylamines. However, initial SAR investigations of a series of 4-alkoxysubstituted 3,5-dimethoxyphenethylamines and their α-methyl congeners revealed a similar trend in that more lipophilic 4-substituents lead to higher affinities (Parker et al., 2008; Trachsel et al., 2013). Similarly, 3,5-dimethoxy derivatives with more lipophilic 4-substituents also lead to more potent compounds in man, when not surpassing a certain steric bulkiness (Shulgin and Shulgin 1991; Trachsel et al., 2013).

#### 4.1.1 5-HT_1A_ Receptor Binding

In the present investigation, only a few phenethylamine derivatives (MDFM; **11**, DFM; **12**, TFM; **13**, CP; **29**, MAL; **32**, and BZ; **33**) slightly augmented the binding affinity at the 5-HT_1A_ receptor when compared to mescaline (**5**). None of the α-Me-containing compounds showed affinities at this receptor subtype (*K*_i_ > 5,600 nM), indicating that the 5-HT_1A_ receptor does not tolerate this steric expansion in 3,4,5-trisubstituted amphetamines. This is in line with other α-Me-containing compounds like 2,4,5-trisubstituted amphetamines (Kolaczynska et al., 2019).

#### 4.1.2 5-HT_2A_ Receptor Binding

All tested phenethylamine derivatives, except for DFIP (**23**), displayed an increased affinity at the 5-HT_2A_ receptor compared to mescaline (**5**). However, these 5-HT_2A_ receptor interactions were less potent when compared to other psychedelic phenethylamines (for instance NBOMe or 2,4,5-trisubstituted derivatives), which bind in the low nanomolar range (Rickli et al., 2015; Luethi et al., 2018b; Kolaczynska et al., 2019; Luethi et al., 2019). This is in line with what has been observed so far for 3,4,5-trisubstituted phenethylamines (Monte et al., 1997; Trachsel et al., 2013). Nowadays, it is well established that phenethylamine psychedelics induce their psychoactive effects mainly by agonistic action at the 5-HT_2A_ receptor (Glennon et al., 1992; Monte et al., 1996; Chambers et al., 2001; Chambers et al., 2002). However, different downstream signaling cascades, biased agonism, and other pharmacological targets may contribute to the subjective effects. Mescaline (**5**) binds and activates the 5-HT_2A_ receptor as partial agonist with low potency *in vitro* (Monte et al., 1997; Rickli et al., 2016). Nevertheless, *in vivo*, it induces intense and long lasting psychedelic effects if applied at high doses (Shulgin and Shulgin 1991). This suggests that low affinity binding to the receptor does not exclude marked psychoactivity *in vivo* when the corresponding compound is ingested at an adequate dose*.* In fact, it has been shown that binding affinity serves as a marker of the clinical doses needed to induce such effects (Luethi and Liechti 2018). However, for 3,4,5-substituted derivatives additional pharmacological interactions or targets may significantly contribute to the overall psychedelic effects observed in humans. Furthermore, it is important to note that 5-HT_2A_ agonists have a higher apparent affinity for receptors labeled with an agonist as displacement ligand compared to an antagonist displacement ligand (Sleight et al., 1996). Therefore, the apparent affinity of 3,4,5-substituted phenethylamines and amphetamines for the 5-HT_2A_ receptor depends on the intrinsic efficacy of the radioligand used. This complicates the correlation of *K*_i_ values, which were assessed using an antagonistic labelling setup, with psychoactive doses.

The most promising modifications resulting in increased affinity at the 5-HT_2A_ receptor were 4-trifluoromethoxy (TFM, **13**), 4-methallyloxy (MAL, **32**), and 4-benzyloxy (BZ, **33**) substituents, resulting in 17- to 63-fold higher affinities. The aforementioned derivatives except for **33** are known to be active in humans and show up to 9-fold higher potency when compared to **5** (Shulgin and Shulgin 1991; Trachsel 2002; Trachsel 2003; Trachsel et al., 2013). Since the amphetamine homolog 3C-BZ induces psychedelic effects similar to LSD (**3**) or TMA (**6**) (Shulgin and Shulgin 1991), BZ (**33**) may induce psychedelic effects as well, based on its similar structure and high binding affinity at the 5-HT_2A_ receptor.

Similar to the investigated phenethylamines, all examined structural modifications on the amphetamine derivatives increased affinity at the 5-HT_2A_ receptor when compared to TMA (**6**). Most derivatives bound in the micromolar range and showed at least a 3-fold increase in 5-HT_2A_ receptor affinity compared with **6**. 3C-P (4-propyloxy substituent; **28**) and 3C-AL (4-allyloxy substituent; **31**) were the most potent amphetamine derivatives, showing at least a 10-fold increase in 5-HT_2A_ receptor affinity, equivalent to the binding observed for highly potent phenethylamine derivatives such as TFE (**18**) (Trachsel et al., 2013). The phenethylamine analogs of **28** and **31**, namely proscaline (**24**) and AL (**30**), respectively, are among the most potent phenethylamines in the 3,4,5-series (Shulgin and Shulgin 1991). Previous research and the present study suggest that α-methyl containing congers bind with slightly higher affinity to the 5-HT_2A_ receptor and show slightly greater activation potency (Shulgin and Shulgin 1991; Trachsel et al., 2013). This would suggest **28** and 3C-AL (**31**) to be relatively potent psychedelics. In fact, it has been reported that **31** is active in humans with doses lying in the range of 15–30 mg (Trachsel et al., 2013).

#### 4.1.3 5-HT_2C_ Receptor Binding

Similar to the 5-HT_2A_ receptor binding, all phenethylamine derivatives, except for DFIP (**23**), had substituents that improved the affinity at the 5-HT_2C_ receptor (*K*_i_ = 290–5,700 nM) when compared to **5** (*K*_i_ = 9,900 nM). The increase in 5-HT_2C_ receptor affinity compared to **5** was 2- to 8-fold for most derivatives. Exceptions were TFM (**13**), MAL (**32**), and BZ (**33**), which displayed submicromolar affinity at the 5-HT_2C_ receptor (*K*_i_ = 290–520 nM), with 19- to 34-fold higher affinity than **5**. Similarly, the amphetamine derivatives showed increased binding to the 5-HT_2C_ receptor (*K*_i_ = 1,700–8,400 nM) compared to TMA (**6**) (*K*_i_ > 10,000 nM).

#### 4.1.4 5-HT Receptor Subtype Selectivity

Most of the 3,4,5-substituted phenethylamine and amphetamine derivatives had moderate to high preference for the 5-HT_2A_ over the 5-HT_1A_ receptor (up to 29-fold 5-HT_2A_ vs 5-HT_1A_ binding ratio), similar to psychedelic 2C derivatives investigated earlier (Rickli et al., 2015; Luethi et al., 2018b; Kolaczynska et al., 2019; Luethi et al., 2019). A minority of the substances were either slightly more selective for the 5-HT_1A_ receptor or non-selective.

Overall, the tested derivatives showed similar affinities at the 5-HT_2A_ and 5-HT_2C_ receptors, with some compounds being slightly more selective for one or the other receptor subtype. This is not uncommon and has been observed for most of the many investigated ligands with a substituted phenethylamine or amphetamine pharmacophore in past (Glennon et al., 1992; Monte et al., 1996; Chambers et al., 2001; Chambers et al., 2002). Notably though, based on extensive SAR investigations, a few agonists with a remarkable 5-HT_2A_ vs 5-HT_2C_ receptor selectivity have been designed. However, these compounds were not simple phenethylamines but a conformationally restricted phenethylamine derivative (2A vs 2C selectivity of 124) (Juncosa et al., 2013) and a *N*-(2-hydroxybenzyl) substituted phenethylamine (2A vs 2C selectivity in the range of 52–81, depending on the assay type) (Jensen et al., 2017). Both 5-HT_2A_ and 5-HT_2C_ receptor affinities have been shown to correlate with clinical potency of psychedelics (Luethi and Liechti 2018). However, assessed affinity values and observed trends might potentially differ if instead of using antagonists, agonists would be used as displacement ligands (Sleight et al., 1996; Colom et al., 2019). Moreover, 5-HT_2A/2C_ interactions are not the only factors that influence potency in humans and pharmacokinetics may potentially have a significant impact on *in vivo* effects. Namely, interactions with other monoamine receptors, lipophilicity, receptor activation, functional selectivity, and metabolism *via* cytochrome P450 enzymes or amine oxidases could also play a role.

In general, we observed the following SAR in regards to affinity at the investigated 5-HT receptor subtypes: an extension of the carbon chain or fluorination of the 4-alkyloxy moiety in the 3,4,5-substituted series moderately increased the binding affinity at the 5-HT_1A_ receptor for some phenethylamine derivatives. This effect was previously observed with 4-alkoxy substituted 2,5-dimethoxyphenethylamines and 2,5-dimethoxyamphetamines (Kolaczynska et al., 2019), where similar structural modifications had little effect on the 5-HT_1A_ receptor affinity. In contrast and in line with previous studies, extension of the carbon chain at the 4-alkyloxy moiety enhanced binding affinity for the derivatives tested within the scope of this study (Dowd et al., 2000; Luethi et al., 2018b; Kolaczynska et al., 2019). The number of fluorine atoms at the 4-alkyloxy moiety proportionally increased the binding affinity at the 5-HT_2A_ and 5-HT_2C_ receptor (e.g., affinities at the 5-HT_2A_ receptor; mescaline *K*_i_ = 9,400 nM > DFM *K*_i_ = 3,500 nM > TFM *K*_i_ = 280 nM). The presence of an α-Me group had only little and mixed effects on the compounds with the same substituents (mescaline [**5**] vs TMA [**6**], FP [**25**] vs 3C-FP [**27**], FE [**16**] vs 3C-FE [**20**], or DFE [**17**] vs 3C-DFE [**19**]). A previous investigation of some of the herein investigated derivatives revealed that introduction of an α-Me group causes slight increases in binding affinity at the 5-HT_2A_ but not 5-HT_2C_ receptors (Trachsel et al., 2013). Affinities assessed in the present study differ slightly from previously reported data, likely explained by differences in used assays and cell lines (Trachsel et al., 2013).

### 4.2 Activation Potency and Efficacy at the 5-HT_2A_ and 5-HT_2B_ Receptors

The derivatives with high 5-HT_2A_ receptor affinities (*K*_i_ < 1,000 nM), such as TFM (**13**), MAL (**32**), and BZ (**33**), also displayed high activation potency (EC_50_ in the range of 27–280 nM). Structures **13**, and **33** were found to be partial agonists (efficacy < 85%) and **32** had an activation efficacy of 85%, suggesting full agonist properties. In accordance to these *in vitro* findings, potent psychedelic effects have been described for TFM (**13**) and MAL (**32**) (Shulgin and Shulgin 1991; Trachsel et al., 2013), suggesting **33** to be potentially psychedelic in humans. The remaining substances were less potent partial to full 5-HT_2A_ agonists. However, for various substances a discrepancy between binding and activation was observed (i.e., activation potency was distinctively higher than affinity). It has previously been described that unlike receptor binding values, activation potency assessed with a Ca^2+^ mobilization assay does not necessarily correlate with the potency of the drug (Luethi and Liechti 2018). Functional assays based on other signaling events, for instance IP formation or β-arrestin recruitment, might better predict the clinical potency of scalines and 3C-scalines. In addition to 5-HT_2A/2C_ receptor activity, the head twitch response is an established method to predict the activity and potency of psychedelics (Halberstadt et al., 2011; Halberstadt and Geyer 2014; Halberstadt et al., 2019; Halberstadt et al., 2020). Halberstadt et al. (2019) recently showed that 3C-E (**21**) and 3C-P (**28**) induced a head twitch response with almost identical potency. Thus, **28** may induce psychedelic effects in humans at similar doses as **21** (Halberstadt et al., 2019).

Among all tested substances, the only potent partial 5-HT_2B_ agonists were the phenethylamine derivatives MDFM (**11**), TFM (**13**), and TFE (**18**) (EC_50_ of 88–210 nM), and the amphetamine derivatives 3C-DFM (**14**), 3C-DFE (**19**), 3C-FE (**20**), and 3C-E (**21**) (95–800 nM). However, these substances were low efficacy partial agonists (EC_50_ = 18–45%). Endocardial fibrosis has been associated with 5-HT_2B_ activation and is therefore a potential adverse effect to consider for chronic use of substances interacting with this receptor (Rothman et al., 2000; Droogmans et al., 2007; Roth 2007; Doly et al., 2008; Elangbam et al., 2008; Bhattacharyya et al., 2009; Huang et al., 2009; Elangbam 2010; Dawson and Moffatt 2012). As psychedelics are typically not used chronically, endocardial fibrosis is an unlikely adverse effect for users of such substances despite a potential interaction with the 5-HT_2B_ receptor subtype (Luethi et al., 2021).

### 4.3 Non-Serotonergic Monoamine Receptor and Transporter Binding Interactions

None of the investigated phenethylamine and amphetamine derivatives interacted with the human TAAR1, the D_2_ receptor, or monoamine uptake transporters. It is unclear, however, whether co-expression of different receptors would alter a substance’s response at these targets. Still, some derivatives bound to the rat TAAR1 with moderate to high affinity and some substances additionally showed low affinity at the mouse TAAR1. These results confirm the previously observed TAAR1 affinity rank order (rat > mouse > human TAAR1) (Wainscott et al., 2007; Lewin et al., 2008; Rickli et al., 2015; Simmler et al., 2016; Luethi et al., 2018b; Kolaczynska et al., 2019; Luethi et al., 2019). TAAR1 has been shown to negatively modulate monoaminergic neurotransmission (Lindemann et al., 2008; Revel et al., 2011) but the lack of human TAAR1 activation calls into question the relevance of TAAR1 in the mechanism of action of scalines and 3C-scalines. All phenethylamines moderately to weakly interacted with the α_2A_ receptor (*K*_i_ = 450–3,700 nM) but only MDFM (**11**) and TFM (**13**) bound to the α_1A_ receptor (*K*_i_ = 3,200–4,300 nM). Among the amphetamines, only TMA (**6**), 3C-DFM (**14**), and 3C-P (**28**) bound to the α_2A_ receptor (*K*_i_ = 2,600–4,600 nM) whereas no binding to the α_1A_ receptor was observed. This is in line with a previously reported higher α_2A_ vs α_1A_ receptor selectivity observed for psychedelic 2,4,5-substituted phenethylamines (2C derivatives) (Rickli et al., 2015; Luethi et al., 2018b; Kolaczynska et al., 2019; Luethi et al., 2019). As observed for the 5-HT_2A_ and 5-HT_2C_ receptors, binding affinity at non-serotonergic receptors increased proportionally to the number of fluorine atoms (e.g., affinities at rTAAR1: mescaline *K*_i_ = 3,000 nM > DFM *K*_i_ = 880 nM > TFM *K*_i_ = 170 nM; DFE *K*_i_ = 2,100 nM > TFE *K*_i_ = 1,200 nM; FP *K*_i_ = 1,700 nM > TFP *K*_i_ = 910 nM).

## 5 Conclusion

In the present investigation, we pharmacologically examined a series of 4-alkoxy-substituted 3,5-dimethoxyphenethylamines (scalines) and 4-alkoxy-substituted 3,5-dimethoxy-amphetamines (3C-scalines) *in vitro*. Psychedelic activity in humans has been reported for several of the tested compounds but detailed information on their monoaminergic interactions was hitherto lacking. Overall, the tested compounds interacted from moderate to high potency with the 5-HT_2A_ receptors and to a slightly lesser extent, with the 5-HT_2C_ receptors. Additionally, various compounds bound to adrenergic α_1A_ and α_2A_ receptors, which may therefore modulate the pharmacodynamics together with serotonergic receptor activation. Compared to mescaline (**5**), various structural modifications of the 4-alkoxy substituent, including introduction of fluorine substituents, increased the 5-HT_2A_ and 5-HT_2C_ receptor affinities. Mescaline (**5**) has recently regained interest as therapeutic agent in psychiatry. The results of the present study suggest therapeutic potential for several novel mescaline derivatives as well.

## Data Availability

The original contributions presented in the study are included in the article/Supplementary Material, further inquiries can be directed to the corresponding author.
